# Assessing the risk of *Staphylococcus aureus* contamination and occupational exposure on high-frequency contact surfaces in funeral venues

**DOI:** 10.3389/fpubh.2026.1823786

**Published:** 2026-06-05

**Authors:** Pan Sun, Peiru Xu, Li Chen, Fang Chen, Bin Zhang, Lei Wu

**Affiliations:** 1 Disinfection and Vector Control Institute, Anhui Provincial Center for Disease Control and Prevention, Hefei, Anhui, China; 2 Antibiotics Laboratory, Anhui Institute for Food and Drug Control, Hefei, Anhui, China

**Keywords:** funeral home, high-touch surfaces, infection control, occupational exposure, quantitative microbial risk assessment (QMRA), *staphylococcus aureus*

## Abstract

**Background:**

Funeral industry workers face potential microbial exposure when handling corpses, yet quantitative risk assessments for specific pathogens like *Staphylococcus aureus* remain limited.

**Objective:**

This study assessed health risks to funeral staff from *S. aureus* [including methicillin-resistant *Staphylococcus aureus* (MRSA)] exposure via contaminated surfaces, identifying critical risk nodes and contributing factors. Methods: In 2024, we collected 85 swab samples from high-touch surfaces (cosmetics, tools, faucets, door handles, countertops) across four Anhui funeral parlors. Total bacterial counts, coliforms, and *S. aureus* were quantified. Using on-site data and occupational parameters, we developed a quantitative microbial risk assessment (QMRA) model for the “surface-hand-mouth” transmission route, employing Monte Carlo simulation (10,000 iterations) and comparative risk ranking.

**Results:**

Surface total colony count qualification rate was 91.76%, with *S. aureus* detected on 16.47% of surfaces; 35.71% of isolates were MRSA. Faucets showed highest *S. aureus* concentrations. The median potential intake via indirect environmental contact (faucets: 146.7 MPN/day) was 2.15 times higher than direct contact with makeup tools (68.2 MPN/day). Sensitivity analysis identified hand-mouth transfer efficiency and faucet contamination levels as key uncertainty drivers.

**Conclusion:**

This first-ever combination of environmental monitoring and quantitative exposure modeling in funeral hygiene reveals that indirect environmental exposure may pose greater *S. aureus* transmission risk than direct tool contact. Findings emphasize enhanced cleaning of high-touch surfaces and rigorous hand hygiene, providing scientific basis for risk-based infection control strategies.

## 1 Introduction

Funeral venues, as specialized public service nodes for handling remains, pose underestimated environmental health risks within the public health system (1, 2). Bodies, particularly those of individuals who died from infectious diseases, serve as complex sources of biological pollution. Research indicates that 24–48 h post-mortem, microorganisms within the body, including various opportunistic pathogenic bacteria and pathogens, proliferate significantly due to immune system failure and may be released into the environment through fluid exudation (3). Consequently, funeral workers involved in tasks such as body transportation, embalming, cosmetic surgery, and cremation face continuous and direct occupational exposure to these microorganisms (4, 5). In contemporary China, the annual death toll is substantial, reaching approximately 10.93 million in 2024 (6). Within this total, nearly 25,500 deaths were directly attributed to notifiable infectious diseases (excluding COVID-19) in the same year (7). The resulting bodies requiring funeral services have placed a spotlight on infection control within the industry (8). Thus, funeral parlors potentially act as a “last line of defense” in preventing the spread of infectious diseases from medical institutions to communities (1, 8). The environmental hygiene and infection control standards of funeral parlors hold considerable public health importance. Despite advancements, significant gaps remain in understanding the risks associated with funeral venues. Current research predominantly addresses air chemical pollutants or broadly analyzes microbial communities such as total bacteria, coliforms, and environmental fungi (3, 9), but lacks quantitative studies focused on specific pathogens, particularly those posing high occupational health risks like *S. aureus*. Beck-Sague et al. (10) surveyed 860 funeral workers and found that 89 out of 542 respondents (17%) reported occupationally acquired infections, including 27 cases of staphylococcal infections, highlighting the substantial health threats from occupational exposure. Moreover, Zhou et al. (11) reported that the hand carriage rate of pathogenic and opportunistic bacteria (including but not limited to *S. aureus*) among funeral workers was 31.96% (570 out of 1,783 isolated strains), with embalmers/cosmetologists showing the highest rate at 35.67%. This is comparable to or even higher than the estimated 21%−25% nasal carriage rate of *S. aureus* in the general population (12, 13), suggesting that occupational exposure may not simply elevate colonization but still poses infection risks via contaminated hands. Thus, there is an urgent need to shift research from “widespread environmental pollution” to “occupational exposure risk assessment targeting specific pathogens” to accurately identify risks and develop effective prevention and control strategies.

In occupational exposure pathways, high-frequency contact surfaces act as crucial links between pollution sources (such as remains and their contaminated environments) and vulnerable hosts, like workers (14). Commonly touched areas, including door handles, device buttons, and tool surfaces, can temporarily harbor pathogens and facilitate their transmission (15). Numerous studies have demonstrated that various pathogens, ranging from methicillin-resistant *Staphylococcus aureus* (MRSA) to norovirus, can persist on different material surfaces for hours to days, spreading efficiently through hand contact (16–19). In hospital infection control, the contamination level of these high-contact surfaces is a key indicator for assessing infection risk and the effectiveness of interventions (20). However, in high-risk environments like funeral venues, systematic research on the pathogen contamination of high-contact surfaces is nearly absent.

The current research gaps are primarily concentrated in three areas. Initially, in funeral settings, there is a lack of quantitative research on *S. aureus* and MRSA. Most studies focus only on total colony counts or microbial community composition (2, 21), neglecting the identification of specific pathogens like *S. aureus* that pose significant health risks to practitioners. This oversight results in inadequate risk assessment and insufficient warnings. Additionally, the assessment methods are overly simplistic. Without a risk assessment model that integrates quantitative data on environmental microorganisms with occupational exposure factors, such as exposure frequency and hand hygiene practices, transitioning from “detecting contamination” to “quantifying risk” remains challenging. Although the quantitative microbial risk assessment (QMRA) framework is well-established in other fields (22, 23), it has yet to be applied to funeral hygiene. Finally, the hygiene indication system is incomplete. While total colony counts reflect general hygiene conditions, they fail to indicate specific transmission routes. For fecal contamination, coliform bacteria are appropriate indicators. However, for assessing hand hygiene, cross-contamination, and indirect contact transmission on high-touch surfaces, *S. aureus*—although not primarily fecal-associated—serves as a relevant hygiene indicator (24). Its presence on surfaces reflects human shedding, inadequate handwashing, and the potential for occupational exposure. Therefore, incorporating both coliforms (for fecal contamination) and *S. aureus* (for contact-related contamination) can more comprehensively identify weaknesses in environmental hygiene management.

Addressing the identified research gaps, this study uses a funeral home in Anhui Province, China, as a case study to assess microbial contamination with a focus on occupational exposure risks. The primary aim is to move beyond merely describing contamination to quantifying and classifying the associated risks.

This study aims to achieve several objectives: First, it seeks to gather background data on pollution by quantitatively assessing contamination levels of *S. aureus* at critical locations and frequently touched surfaces in funeral venues through on-site sampling. Second, it involves constructing and implementing exposure assessment models. These models, based on actual working scenarios and behavioral patterns, quantitatively assess microbial exposure by calculating potential exposure doses for personnel in various positions using the collected pollution data. Finally, the study compares and ranks risk pathways. By employing a comparative risk assessment method, it quantitatively evaluates and ranks the exposure risks associated with different work processes and contact pathways, identifying key exposure nodes that require prioritized control.

The theoretical significance of this study is that it systematically introduces and applies a QMRA framework, transitioning from pollution monitoring to dose modeling within the realm of funeral occupational health. This approach allows for the quantitative description and comparison of occupational exposure risks to specific pathogens, offering a replicable methodological path for risk assessment in this field. Practically, the study's findings provide a direct basis for precise infection control management in funeral venues. They offer data support for implementing and optimizing relevant standards and help formulate targeted intervention strategies for high-risk positions and exposure routes, thereby more effectively safeguarding the occupational health of practitioners.

## 2 Method

### 2.1 Research design and site

This study employs a QMRA framework to evaluate the occupational health risks faced by staff in funeral facilities exposed to *S. aureus* through contact with frequently contaminated surfaces. The research combines cross-sectional surveys with mathematical model simulations. Initially, background data on microbial contamination of key surfaces were gathered through on-site sampling. Using this data as a crucial input parameter, an exposure assessment model reflecting real working scenarios was developed. Finally, a comparative risk assessment method was used to rank the risks associated with different exposure routes.

The research, conducted in 2024, employed a purposeful sampling method. Four funeral facilities of varying operational scales, including large urban and regional-level sites, were chosen within Anhui Province for the survey. The study primarily concentrates on the key surfaces that staff frequently contact during their daily tasks.

### 2.2 Sample collection and microbiological testing

#### 2.2.1 Sampling objects and methods

The target surfaces in funeral venues include those frequently touched by staff during their work, such as makeup and cosmetic tools, embalming operation surfaces, faucet switches in the operation room, and various handles (including door, refrigerator, and stretcher cart handles). Surface sampling was performed following the procedures specified in MZ/T 140-2019 (the funeral industry standard for pathogenic bacteria detection) (25) and GB 19053-2024 (safety limits for pathogenic bacteria in funeral venues) (26). A sterile cotton swab moistened with phosphate-buffered saline (PBS) containing a neutralizer was rubbed evenly over each target surface within a 5 cm × 5 cm sterile template. Samples were stored at 4 °C and transported to the laboratory for analysis within 2 h of collection.

#### 2.2.2 Microbiological detection and analysis

Each sample was subjected to the following tests, and the results were directly incorporated into the risk assessment model:

Total viable bacteria count: Enumeration of total viable bacteria was performed using the pour plate method as described in MZ/T 140-2019 (25) and GB 19053-2024 (26). Samples were plated on nutrient agar (NA) and incubated at 37 °C for 48 h. After incubation, all visible colonies were counted manually, and results were expressed as colony-forming units per square centimeter (CFU/cm^2^).

Quantification and identification of *S. aureus* was quantified using a three-tube most probable number (MPN) method according to MZ/T 140-2019 (25). Baird-Parker agar was used for selective screening. Presumptive colonies were subcultured and identified using the VITEK 2 Compact system. MRSA was detected by the cefoxitin disk diffusion method (30 μg disk). The detection limit of the MPN procedure was 0.3 MPN/cm^2^ over the sampled surface area. Results are expressed as MPN/cm^2^.

Enumeration of coliforms (indicator of fecal contamination): Coliforms were enumerated using the lauryl tryptose MPN (LTMP) fermentation method at 37 °C according to MZ/T 140-2019 (25) and GB 19053-2024 (26). Positive presumptive tubes were confirmed by streaking onto eosin methylene blue (EMB) agars, and confirmed isolates were identified using standard biochemical tests. In the context of funeral venues, the presence of coliforms serves as a strong indicator of environmental fecal contamination.

### 2.3 QMRA framework

This study employs a QMRA (QMRA) framework. Methodologically, it draws on the construction concepts and uncertainty analysis techniques used by Overbey et al. (27) to assess the risk of norovirus transmission on surfaces in medical settings. The study follows the internationally recognized QMRA standard process: hazard identification, exposure assessment, and risk characterization, with adjustments for the specific pathogen, *S. aureus*, and the unique context of funeral venues. In hazard identification, *S. aureus* is identified as the target pathogen, with particular attention to the additional treatment challenges and public health risks posed by its methicillin-resistant strains. For exposure assessment, a mathematical model of the “surface-hand-mouth” exposure pathway is developed to quantify the potential microbial dose funeral facility staff may ingest daily through contact with contaminated surfaces. The exposure modeling approach was adapted from Overbey et al. (27), and the multi-step transfer analytical methods from Julian et al. (29), the transfer efficiency parameters from Rusin et al. (28), and the QMRA application parameters from Ryan et al. (30). In risk characterization, due to the absence of a recognized dose-response relationship model for oral *S. aureus* infection, the study employs a “comparative risk assessment method.” This method ranks the risk levels of different work activities by calculating a relative risk index, representing an adaptive adjustment to the standard QMRA process in light of data limitations.

#### 2.3.1 Model scenario definition

**Figure 1:**
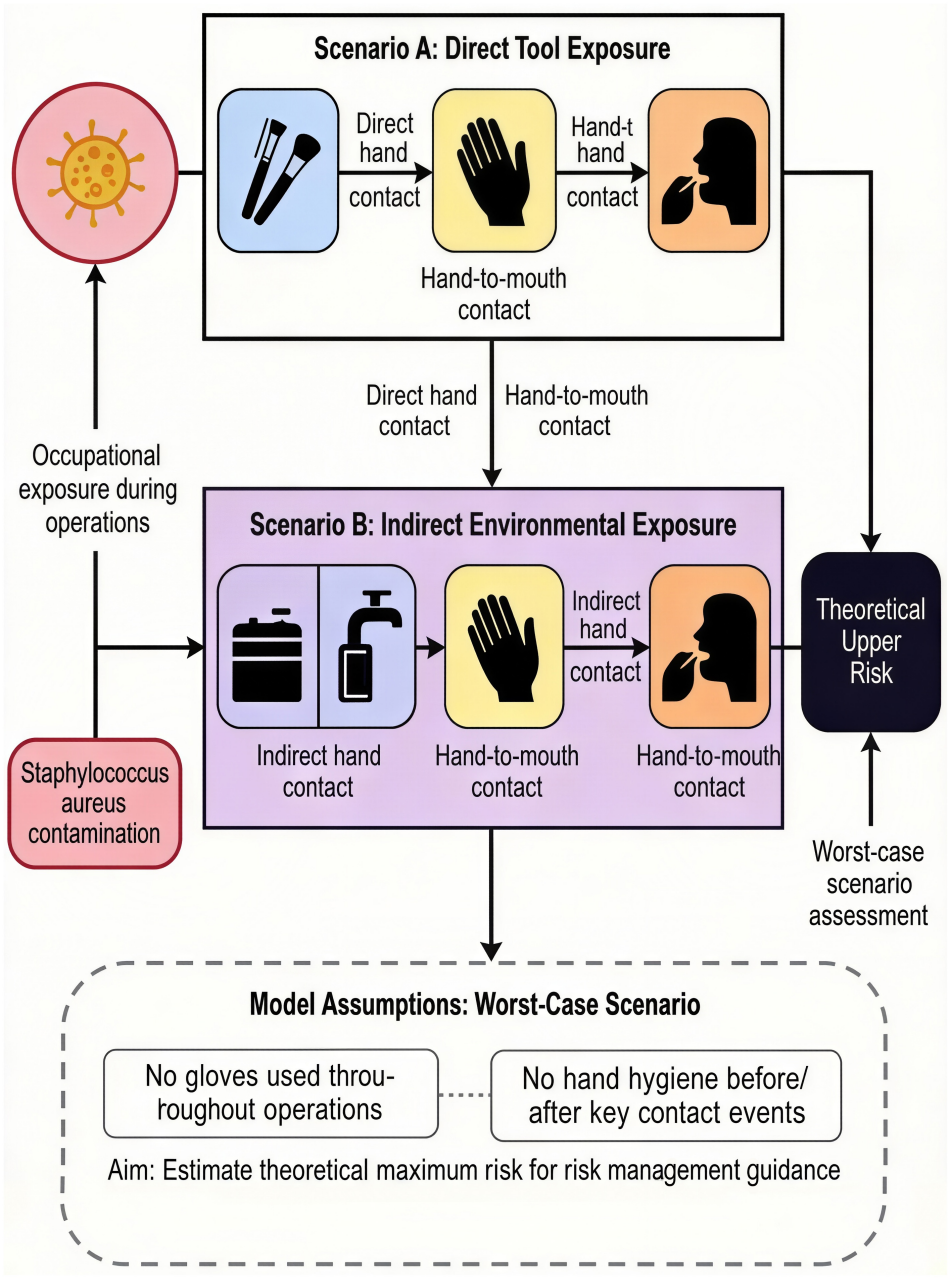
Flowchart of the two primary exposure scenarios for funeral site staff.

To accurately simulate real occupational exposure, this study outlines two primary exposure scenarios for funeral site staff (Figure 1)

Scene A (Direct Exposure to Tools): during operation, individuals directly handle makeup tools contaminated with *S. aureus* with their bare hands, subsequently leading to hand-to-mouth contact.

Scenario B (Indirect exposure to the environment): during or after an operation, an individual makes bare-handed contact with a contaminated workbench, handle, or faucet, and subsequently touches their mouth.

To evaluate the theoretical upper limit of risk, the model was configured as a “worst-case scenario”: no personal protective gloves were worn during the operation, and no hand cleaning or disinfection measures were implemented before or after the critical contact event. This assumption is intended to determine the “theoretical risk ceiling” or “potential maximum risk,” thereby providing a foundation for subsequent risk management recommendations.

#### 2.3.2 Exposure dose estimation model

To estimate the potential daily intake of *S. aureus* by funeral facility staff via the “surface-hand-mouth” route, the model employs the following calculation sequence:

Amount of contamination per contact: the number of pathogens transferred from the surface to the hands during a single contact by staff with bare hands, denoted as *Ns*,*per*–*event* (MPN/time), is calculated using the formula:


$$Ns,per-event=Cs\;\times \;Acontact\times \;\eta_{s-h}$$

Among them:

*Cs*: the contamination concentration of *S. aureus* on surface ^*^s^*^ (MPN/cm^2^) was represented by the median value of the detection results in this study, which served as the model input.

*Acontact*: the effective contact area, measured in square centimeters (cm^2^), between the hand and the surface of an object during a single contact.

η_*s*−*h*_: This term refers to the transfer efficiency, or proportion, of microorganisms moving from an object's surface to wet hands.

Average daily hand contamination dose: taking into account the frequency of daily contact, the average daily total hand contamination dose, denoted as Dhand, resulting from contact with surface ^*^s^*^, *s* (MPN/day) is:


$$Dhand,s=Ns,per-event\times Fs=Cs\times Acontact\times \eta_{s-h}\times \;Fs$$

In this context, Fs denotes the estimated daily frequency of unarmed contact with the surface ^*^s^*^.

Average daily potential intake dose: taking into account hand-to-mouth contact behavior, the daily oral potential intake dose, ingestion *D*_*ingestion*_(MPN/day), is calculated by multiplying the total average daily hand contamination dose on each surface by the hand-to-ingestion mouth transfer efficiency:


$$D_{ingestion}=(\sum_{s}Dhand,s)\times \;\eta h-m$$

Here, η_*h*−*m*_denotes the efficiency with which the pathogen is transferred from the hands to the mouth.

Model assumption explanation: the calculation above operates under a “worst-case” assumption, meaning that no personal protective gloves were worn during the operation, and no hand cleaning or disinfection measures were implemented before or after the critical contact event. This assumption is designed to theoretically assess the maximum potential risks, thereby identifying priority areas for risk control and providing a foundation for developing adequate protective measures.

#### 2.3.3 Risk characterization (Comparative Risk Assessment Method) to calculate the maximum

*D*_*ingestion*_ value for all defined scenarios, the scenario with the highest risk is set as the benchmark risk, with a relative risk index of 1.0. The relative risk index for other scenarios is determined using the following formula: RRI_*scenario*_ = *D*_*ingestion, scenario*_ / max (*D*_*ingestion*_).

This index is a dimensionless value ranging from 0 to 1, where a higher value indicates a greater relative risk of exposure.

#### 2.3.4 Model parameters and data sources

The determination, distribution, and scientific basis of model parameters and all input parameters are outlined in Supplementary Table S1. These parameters are established following a transparent and conservative principle, ensuring that risks are not overestimated.

#### 2.3.5 Uncertainty analysis and sensitivity analysis

To address the uncertainty of input parameters in the risk assessment model and quantify their impact on the output, this study employed a comprehensive analysis based on the probability distribution of Monte Carlo simulation. The analysis proceeds as follows:

First, a probabilistic risk assessment is performed to address uncertainties. Key parameters with variability in the model (Supplementary Table S1) are represented as probability distributions to capture real-world uncertainties. Based on this framework, Monte Carlo simulation is used for random sampling calculations.

Secondly, a Monte Carlo simulation is performed using Python 3.13. This involves random sampling of 10,000 independent iterations from the probability distribution of each parameter. For each iteration, a complete set of random parameter values is generated, allowing for the calculation of the corresponding instantaneous exposure dose and daily cumulative exposure dose.

Finally, clarify the analysis content and output: the primary focus of the simulation analysis encompasses:

Conduct an uncertainty analysis by summarizing all iteration results. Report the probability distribution of the exposure dose and the relative risk index (RRI), and characterize these with statistical measures such as the median and its 95% confidence interval.

Sensitivity Analysis: utilizing 10,000 simulation data points, compute the Spearman rank correlation coefficient for each input parameter in relation to the final output (RRI). This process systematically identifies the key driving parameters that most significantly influence the risk assessment results.

### 2.4 Statistics and analysis

The analysis utilized SPSS PRO software. The normality of continuous data was assessed using the Shapiro–Wilk test. Since the data were not normally distributed (*P* < 0.05), medians with interquartile ranges (IQR) were used to describe central tendency and dispersion. Categorical data were presented as frequencies (percentages). The chi-square (χ^2^) test was used for comparing categorical variables. For comparisons of continuous variables across multiple groups, the Kruskal–Wallis *H* test was applied. All tests were two-sided, and a *P* value <0.05 was considered statistically significant.

### 2.5 Quality control and ethics

Both the sampling fluid and culture medium were tested for sterility. Each batch of testing included positive and negative controls. The VITEK 2 Compact system underwent quality control using standard strains prior to use. For the MPN method used to quantify *S. aureus*, the limit of detection (LOD) was 0.3 MPN/cm^2^.

## 3 Results

### 3.1 Overview of microbial contamination on high-frequency contact surfaces

In this study, we collected and analyzed 85 samples from four types of high-frequency contact surfaces in funeral venues. The overall hygiene condition was deemed satisfactory, with a total bacterial count serving as the evaluation index. The overall pass rate was 91.76% (78/85). However, hygiene conditions varied across different surfaces (Supplementary Table S2): door handles (38 pieces) and countertops (10 pieces) achieved the highest pass rates, both at 100%. In contrast, makeup and cosmetic surgery tools (24 sets) had a lower pass rate of 79.17%, while faucets/buttons (13 pieces) had a pass rate of 84.62%.

Analyzing the total bacterial count distribution, the median (P50) for all samples was 0 CFU/25 cm^2^, while the 95th percentile (P95) reached 2,442.40 CFU/25 cm^2^, suggesting some samples were heavily contaminated. Cosmetic and cosmetic surgery tools exhibited the highest contamination, with a median total bacterial count (P50) of 9.40 CFU/25 cm^2^ and a notably uneven distribution (P75: 330.00 CFU/25 cm^2^; P95: 20,712.50 CFU/25 cm^2^). Faucets and buttons had a median bacterial count of 2.60 CFU/25 cm^2^. For door handles, the median total bacterial count was 0 CFU/25 cm^2^ (P75: 1.65 CFU/25 cm^2^), and countertops showed a median of 0.10 CFU/25 cm^2^. Non-parametric tests revealed a statistically significant difference in bacterial count distribution across the four object types (*P* < 0.05), though no significant difference was found in the pass rate (χ^2^ test, *P* = 0.220).

### 3.2 Contamination status of indicator bacteria and pathogenic bacteria

**Figure 2:**
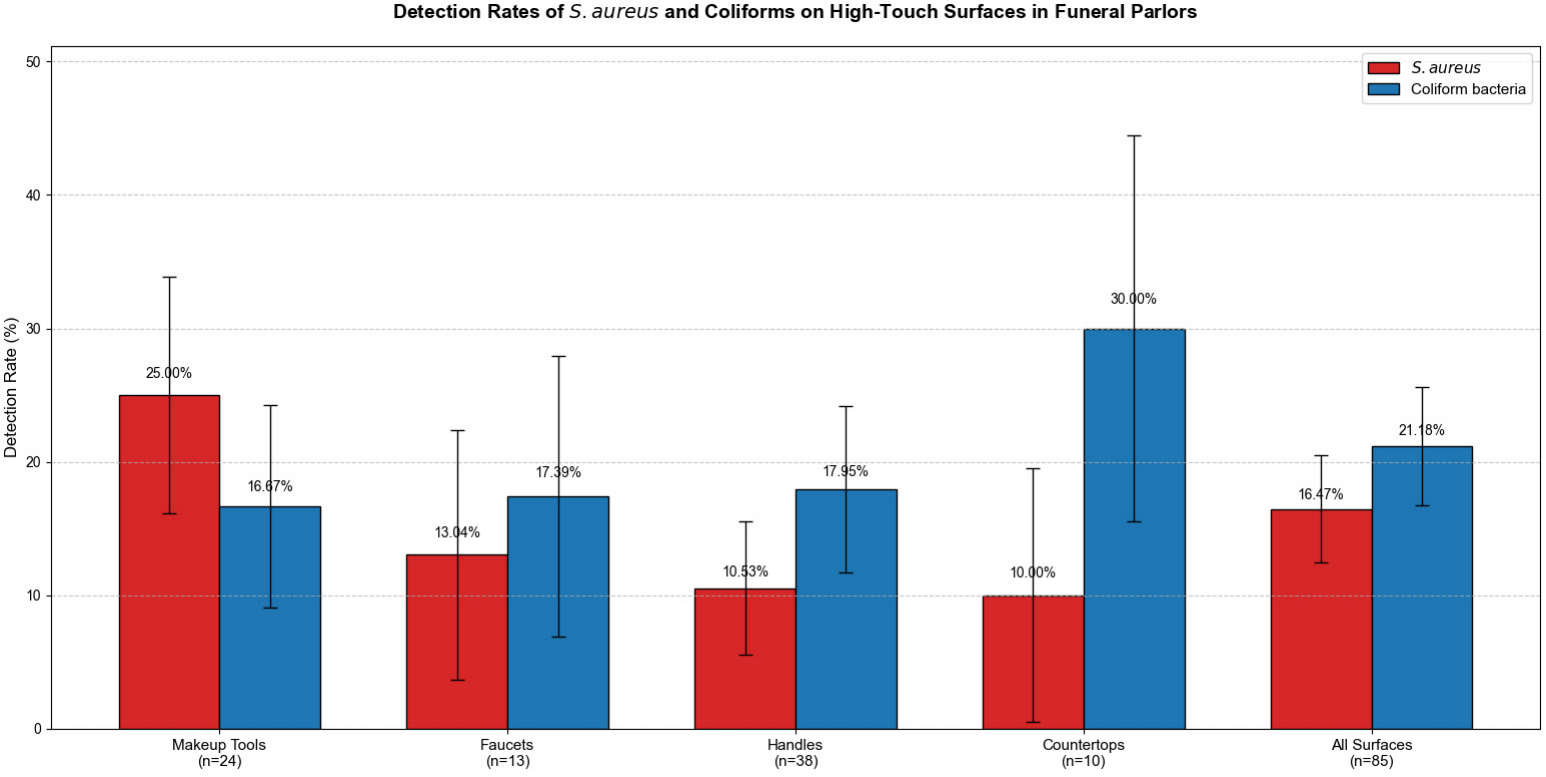
Detection rates of *S. aureus* and coliforms on different high-touch surfaces. Error represent standard error of proportion. Percentages are shown with two decimal places.

Contamination by coliform bacteria and *S. aureus* poses a significant risk. The detection rate for coliform bacteria was 21.18% (18/85), while *S. aureus* was found at a rate of 16.47% (14/85). The contamination patterns for these microorganisms differ (Figure 2): *S. aureus* was most frequently detected in cosmetics and cosmetic tools, with a rate of 25.00%, whereas coliform bacteria were most prevalent on countertops at 30.00%. *S. aureus* contamination was notably higher on faucets, with a median concentration of 23.25 MPN/cm^2^, approximately ten times greater than that on environmental surfaces like door handles and countertops. Of the 14 *S. aureus* strains identified, five were MRSA, as confirmed by the cefoxitin disc method, resulting in a 35.71% MRSA rate among the detected strains.

### 3.3 QMRA results

Using the defined exposure scenarios and parameters outlined in Supplementary Table S1, the potential oral intake dose from occupational exposure was estimated through a Monte Carlo simulation conducted over 10,000 iterations.

In the worst-case scenario (Supplementary Table S3), the median daily potential intake dose from scenario B (indirect environmental exposure) is 146.70 MPN/day, which is approximately 2.15 times greater than that from scenario A (direct tool exposure, 68.20 MPN/day). When scenario B is used as the benchmark risk (relative risk index RRI = 1.0), scenario A has an RRI of 0.47. A probabilistic assessment using parameter probability distribution further confirmed that the median intake dose in scenario B was 146.70 MPN/day (95% CI: 64.80–342.90), significantly higher than in scenario A, which was 68.20 MPN/day (95% CI: 31.50–156.30), with a median RRI of 0.47 (95% CI: 0.22–0.93).

**Figure 3:**
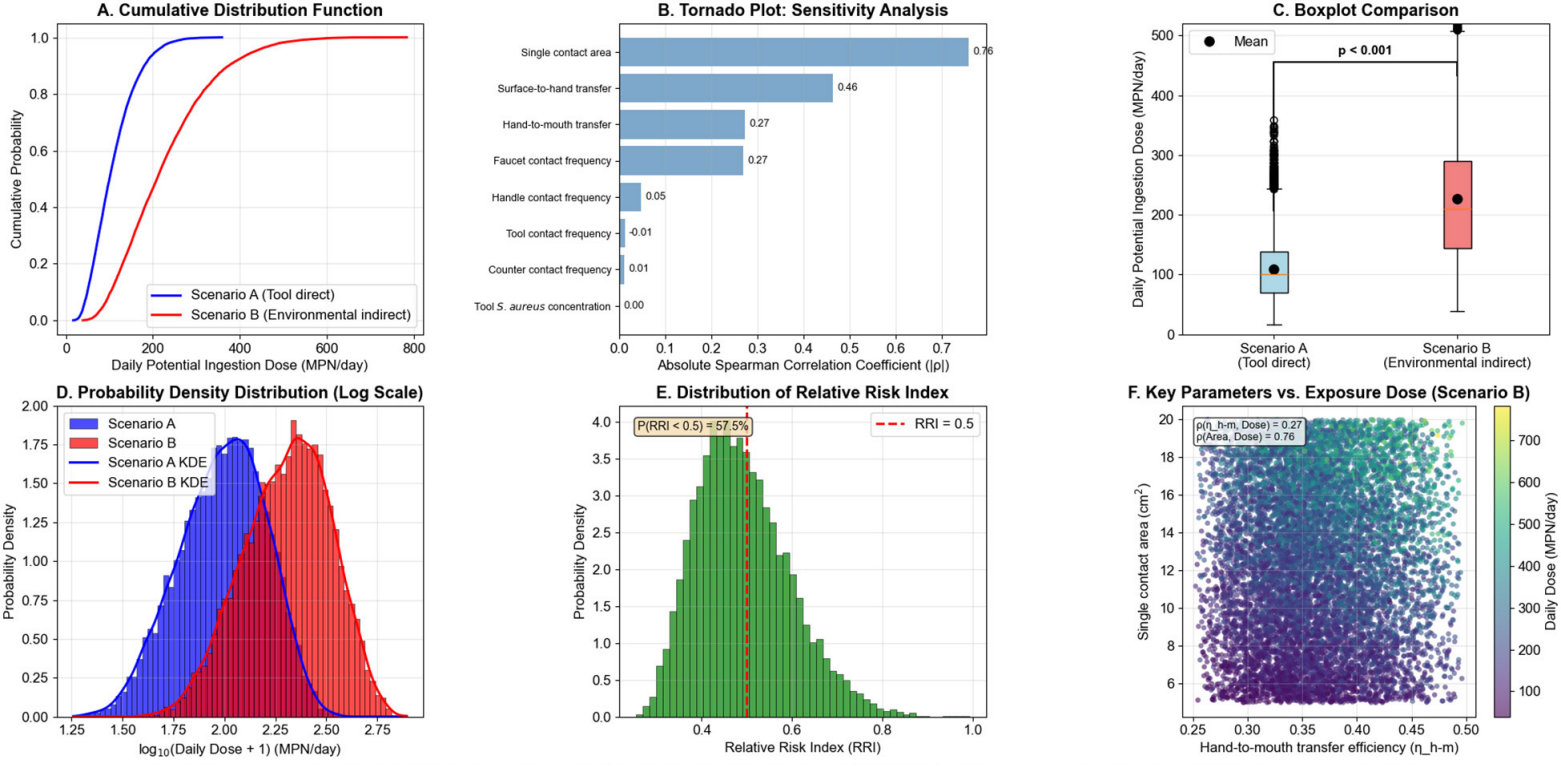
QMRA simulation results. **(A)** Cumulative distribution. **(B)** Sensitivity analysis. **(C)** Boxplot with *p*-value. **(D)** Density on log scale. **(E)** RRI distribution. **(F)** Key parameters vs. dose. Simulations: *n* = 10,000 iterations. *S. auresus* is abbreviated.

As shown in Figure 3, the results of the quantitative risk assessment revealed the distribution characteristics of the exposure dose and the sources of its uncertainty. The sensitivity analysis (Figure 3B) indicated that the uncertainty of the model output was mainly influenced by the hand-to-mouth transfer efficiency (η_*h*−*m*_, ρ = 0.61) and the contamination concentration on the faucet surface (*C*_*faucet*_, ρ = 0.58). This finding was consistent with the correlation pattern between the parameters and the dose in the scatter plot (Figure 3F). Dose distribution analysis further supports the above conclusions. The cumulative distribution function (Figure 3A) and the probability density distribution plot (Figure 3D, log scale) show that the dose distribution of scenario B (indirect environmental exposure) is more dispersed than that of scenario A. The box plot comparison (Figure 3C) confirms that scenario B not only has a higher median intake dose but also a significantly wider 95% confidence interval. The relative risk index distribution (Figure 3E) further quantifies this difference, showing that the median RRI of scenario A is only 0.47 (95% CI: 0.22–0.93). In summary, the variability of input parameters reflected by the Monte Carlo simulation leads to higher uncertainty in the risk estimation of Scenario B (coefficient of variation = 0.57). This uncertainty mainly stems from the spatial heterogeneity of environmental surface contamination levels and the inter-individual variability of practitioners' behavioral parameters (e.g., contact frequency, hand hygiene compliance), highlighting the necessity of implementing standardized operating procedures and regular environmental monitoring in infection control at funeral sites. Quantitative risk assessment shows that under the conditions set in this study, indirect environmental contact (through faucets, doorknobs, etc.) is the main potential route for professional exposure of funeral site staff to *S. aureus*, with a risk approximately twice that of direct contact with tools. This emphasizes the importance of implementing intervention measures such as strict cleaning and disinfection for high - contact environmental surfaces.

## 4 Discussion

This study systematically investigated funeral venues in Anhui Province, combining microbial contamination data from high-frequency contact surfaces with occupational exposure pathways in key positions to develop a QMRA model. For the first time, the results quantified the occupational health risks for funeral facility staff exposed to *S. aureus* via the “surface-hand-mouth” route. More importantly, the study revealed a significant imbalance in risk distribution using a comparative risk assessment method. Key findings include that the risk from indirect contact with environmental surfaces, particularly faucets, is higher than from direct contact with cosmetic tools. Additionally, the study highlighted the compounded effects of behavioral factors, such as hand hygiene, and pathogen characteristics, like MRSA detection, on risk levels. These findings provide a novel scientific basis for implementing precise and hierarchical infection control strategies in this specialized occupational environment.

### 4.1 Pollution characteristics highlight key risk nodes: transitioning from general health indicators to assessing specific occupational hazards

This study revealed that, based on the total number of colonies, the overall hygiene qualification rate of the object surfaces was relatively high at 91.76%, which might suggest that the “overall hygiene condition was good.” However, this broad indicator obscures the specific occupational hazards posed by contamination from particular pathogens. The data indicate that cosmetics and cosmetic tools are among the most hazardous direct-contact surfaces when considering multiple hygiene indicators: they have the lowest total colony count qualification rate at 79.17%, and the median concentration of *S. aureus* contamination is relatively high compared to other surfaces such as door handles and countertops, reaching 5.13 MPN/cm^2^. This is closely linked to their complex structure, difficulty in achieving thorough disinfection, and their operational role involving direct contact with high-microbial-load remains, aligning with the principle that complex instruments pose infection risks in medical environments (31, 32). Notably, the study highlights a key contradiction: tools with frequent direct contact, such as cosmetic and cosmetic surgery tools used 20 times per day, are not the highest-risk pathways in the quantitative model assessment. Instead, environmental surfaces with lower exposure frequencies, like faucets used 8 times a day, present a higher potential exposure risk due to their extremely high median concentration of *S. aureus* contamination at 23.25 MPN/cm^2^, which is more than four times higher than that on cosmetic tools. This finding challenges the simple assumption that “exposure frequency determines risk,” elevating contamination concentration as a decisive factor in risk ranking. It underscores that in the occupational health management of funeral facilities, attention should extend beyond direct operation tools to include frequently touched but potentially overlooked environmental surfaces, such as faucets and door handles, which can be more concealed yet potentially more efficient risk amplifiers.

### 4.2 Development and application of risk assessment models: transitioning from pollution detection to risk quantification

This study advances beyond basic pathogen detection rate reports by introducing, for the first time, a QMRA framework utilizing Monte Carlo simulation within the realm of funeral hygiene. The model integrates environmental monitoring data (pollution concentration) with occupational exposure characteristics (exposure frequency, transfer efficiency), transitioning from merely identifying “presence of pollution” to estimating “possible intake amount.” The assessment results reveal that, under worst-case assumptions, the median daily potential intake dose via indirect environmental exposure (Scenario B) is approximately 2.15 times higher than that from direct contact with tools (Scenario A). This quantitative comparison enables the prioritization of risk across different work processes, highlighting key intervention areas for infection control when resources are limited. Sensitivity analysis further elucidates risk drivers, identifying hand-mouth transfer efficiency (η_*h*−*m*_) and surface contamination concentration (*C*_*faucet*_) as the primary contributors to model output uncertainty. This finding underscores two key points: First, it highlights the critical role of individual behavior, particularly adherence to hand hygiene, in risk prevention and control. Even with high surface contamination levels, proper hand hygiene (reducing η_*h*−*m*_) can effectively interrupt exposure pathways, aligning with hospital infection control conclusions that emphasize the importance of hand hygiene among medical staff in preventing pathogen spread (33). Second, it affirms the importance of environmental control-reducing pollution concentration (*C*_*faucet*_), on key surfaces like faucets is an effective strategy for mitigating overall population risk (34). This insight aligns with the hospital infection control principle that “a clean environment is the foundation of safe medical care,” providing quantitative support for its application in funeral settings (20).

### 4.3 *S. aureus* and MRSA: underrecognized occupational health and public health threats

This study reveals a significant occupational biological hazard in funeral venues, with a *S. aureus* detection rate of 16.47% and high contamination levels on operation-related surfaces. Alarmingly, 35.71% of the detected *S. aureus* strains are MRSA, a proportion comparable to or exceeding that found in some non-critical areas of medical institutions (40, 41). This suggests that funeral venues, particularly corpse-handling areas, might be underestimated hotspots for storing and spreading drug-resistant bacteria outside community and medical settings. These venues contribute to the “thanato-resistome gene pool” (35, 36). While most current research on funeral venues focuses on air chemical pollutants or general microbial communities (3, 37), there is a lack of attention to pathogens with known pathogenicity and drug resistance, such as MRSA (38, 39). Our findings elevate occupational health risks, indicating that workers face exposure not only to pathogens but also to drug-resistant strains with limited treatment options, potentially leading to severe clinical outcomes. Combined with QMRA results, this suggests that funeral facility staff may be continuously exposed to low doses of drug-resistant *S. aureus*, necessitating urgent attention to the long-term health impacts and potential community transmission risks. Therefore, integrating drug resistance monitoring into the routine occupational health monitoring of funeral venues should be a key future focus.

### 4.4 Limitations of the research and future directions

This study has several limitations. A key limitation is the absence of a clear dose-response model for oral *S. aureus*; therefore, we employed a comparative risk assessment approach. Our results focused on risk ranking and identifying key control points rather than estimating absolute infection probabilities. Future research should aim to establish or introduce more comprehensive pathogen-specific dose-response data. Another limitation concerns the source of some model parameters, which were derived from literature in other fields. Their applicability to the specific context of funeral venues—considering factors such as surface materials, temperature, humidity, and organic load—requires validation through further localized studies. Furthermore, the study is based on a “worst-case” assumption (e.g., no glove use or hand hygiene), which may overestimate risks under actual protective measures. Nonetheless, this underscores the critical importance of strictly following existing protective guidelines. In addition, we did not assess whether the workers themselves were nasal or skin carriers of *S. aureus*, a factor that could contribute to surface contamination and represent a potential source of bias. Future studies should include carrier screening to better understand transmission dynamics.

### 4.5 Relevance of ingestion exposure and infectious dose

*S. aureus* is not only a cause of skin and soft tissue infections but also a common agent of toxin-mediated foodborne illness. Ingestion of preformed staphylococcal enterotoxins can lead to rapid onset of vomiting and diarrhea, with an infectious dose (ID50) typically ranging from 105 to 106 CFU or as little as 0.1–1.0 μg of enterotoxin (42). In funeral venues, hand-to-mouth contact after touching contaminated surfaces represents a plausible ingestion exposure route. Although our study focuses on surface-hand-mouth transfer, the potential for toxin-mediated effects—rather than live bacterial infection alone—should be considered in future risk assessments. This further emphasizes the importance of hand hygiene and surface disinfection to reduce both bacterial load and enterotoxin accumulation.

Based on this study's findings, we propose the following targeted risk management strategies: (i) implement hierarchical management by prioritizing environmental surfaces with high pollution risks, such as faucets and door handles, as “core focus” areas for cleaning and disinfection. Set higher disinfection frequencies and standards for these surfaces compared to general ones. (ii) Enhance behavioral interventions. To address the uncertainty of hand-mouth transfer efficiency, improve hand hygiene training, supervision, and compliance assessment for practitioners, particularly those in high-risk roles like preservative cosmetic surgeons. Additionally, provide convenient and effective hand hygiene facilities in key areas. (iii) Initiate drug resistance monitoring. Conduct fixed-point and regular environmental and occupational monitoring of drug-resistant bacteria, such as MRSA, in institutions with the necessary resources to evaluate dynamic changes and the effectiveness of intervention measures. (iv) Implement engineering controls, such as installing foot-pedal-operated sinks, to reduce hand contact with contaminated faucet surfaces, thereby further lowering the risk of indirect environmental exposure.

### 4.5 Conclusion

This study systematically evaluated and quantified, for the first time, the occupational health risks faced by funeral facility staff exposed to *S. aureus* through contaminated surfaces. By integrating on-site microbiological investigations with quantitative risk modeling, the research revealed that indirect environmental exposure may pose a greater risk than direct tool exposure. Surface contamination levels (e.g., *S. aureus* concentrations on high-touch surfaces) and hand hygiene behaviors were identified as key risk drivers. Additionally, the high detection rate of MRSA highlights significant health and safety concerns for those in this occupation. The methodological framework and baseline data from this study offer a new perspective on understanding biological occupational hazards in funeral settings. This study also provides a quantitative scientific basis for implementing and evaluating industry standards and formulating precise infection control strategies based on risk classification. Advancing occupational health protection in funeral venues from general prevention to evidence-based, targeted strategies is essential for safeguarding both practitioners' health and public health security. Future research could extend this QMRA framework to other emerging pathogens of greater public health significance or higher transmission risk, such as Candida auris, to assess occupational exposure risks in funeral settings.

## Data Availability

The raw data supporting the conclusions of this article will be made available by the authors, without undue reservation.
