# Supplementary material for: Assessing the risk of Staphylococcus aureus contamination and occupational exposure on high-frequency contact surfaces in funeral venues
Source: Front Public Health. 2026 Jun 5;14:1823786. doi: 10.3389/fpubh.2026.1823786 (PMC13279306; doi:10.3389/fpubh.2026.1823786)
Supplement: Supplementary file 1 [file Table_1.docx]

**Table 1 Parameter value table**

| **Parameter symbol** | **Parameter description** | **Value and distribution/Value** | **Basis and explanation** |
| --- | --- | --- | --- |
| *N_tool, per-event_* | Hand contamination amount from a single contact with tools | Calculated value | Calculated according to the formula *N_s_*_,_*_per_*_−_*_event_*​=*C_s_*_​_×*A_contact_*_​_×*η_s_*_−_*_h_*_​_ , it is used to estimate the daily average dose. |
| *N_faucet,per-event_* | hand contamination amount from a single contact with faucets | Calculated value | Calculated according to the formula *N_s_*_,_*_per_*_−_*_event_*_​_=*C_s_*​×*A_contact_*_​_×*η_s_*_−_*_h_*_​_ , it is used to estimate the daily average dose. |
| *N_handle,per-event_* | hand contamination amount from a single contact with handles | Calculated value | Calculated according to the formula *N_s_*_,_*_per_*_−_*_event_*​=*C_s_*​×*A_contact_*​×*η_s_*_−_*_h_*_​_ , it is used to estimate the daily average dose. |
| *N_counter, per-event_* | hand contamination amount from a single contact with countertops | Calculated value | Calculated according to the formula *N_s_*_,_*_per_*_−_*_event_*_​_=*C_s_*_​_×*A_contact_*​×*η_s_*_−_*_h_*_​_, it is used to estimate the daily average dose. |
| *D_hand,tool_* | daily average hand dose contaminated via tools | Calculated value | It is calculated by *N_tool,per-event_​*×*F_tool​_* |
| *D_hand,faucet_* | daily average hand dose contaminated via faucets | Calculated value | It is calculated by *N_faucet,per-event_*​×*F_faucet_​* |
| *D_hand, handle_* | daily average hand dose contaminated via handles | Calculated value | It is calculated by *N_handle, per_**_-event_* × *F_handle_* |
| *D_hand,counter_* | daily average hand dose contaminated via countertops | Calculated value | It is calculated by *N_counter, per-event_* × *F_counter_* |
| *C_tool_* | Staphylococcus aureus concentration on the surface of cosmetic tools | 5.13 MPN/cm² | The measured data in this study are from the median of the sampling and testing results of the surfaces of cosmetic tools. |
| *C_faucet_* | Staphylococcus aureus concentration on the surface of faucets | 23.25 MPN/cm² | The measured data in this study. The median of the sampling detection results from the surface of the faucet in the sink of the operation room. |
| *C_handle_* | Staphylococcus aureus concentration on the surface of handles | 1.93 MPN/cm² | The measured data in this study. The median of the sampling detection results from the handle surface. |
| C_counter_ | Staphylococcus aureus concentration on the surface of countertops | 1.85 MPN/cm² | The measured data in this study. The median of the sampling and testing results from the operating table (only one case was detected, and this value was taken). |
| *A_contact_* | single contact area between hands and surfaces | 10 cm^2^ (Uniform distribution: 5 - 20 cm²) | The estimation was made based on the hand size data from the "Handbook of Exposure Parameters for the Chinese Population" and in combination with the analysis of the typical contact patterns between the hands and the countertop/tools during makeup operations. This parameter is a key assumption in the risk assessment model, and its sensitivity can be tested in subsequent analyses. |
| *F_tool_* | daily frequency of bare - handed contact with cosmetic tools | 20 times per day (Triangular distribution: 15, 20, 30) | This parameter is estimated based on the task decomposition analysis of the specific scenario in this study (e.g., the makeup service process). By disassembling the core operation steps (e.g., washing hands before operation, retrieving tools, making adjustments during operation, and cleaning and storing tools after operation) and consulting domain experts, the typical range of the number of times of bare - hand contact with tools was determined. A triangular distribution was used to reflect the uncertainty of this estimation. |
| *F_faucet_* | daily frequency of bare - handed contact with faucets | 8 times per day (Triangular distribution: 5, 8, 12) | This parameter represents the basic frequency of contact with faucets during daily work, independent of hand hygiene practices. It is estimated through observations of workplace activity routes, including initial handwashing upon entering the work area (hypothetical), drinking water, cleaning equipment, and routine water use before and after lunch breaks. A triangular distribution is used to reflect actual variations due to differences in personal habits and work schedules. This setup aims to capture the potential frequency of hand contact with the common surface of faucets, even when additional handwashing due to contamination is not performed. |
| *F_handle_* | daily frequency of bare - handed contact with handles | 12 times per day (Triangular distribution: 8, 12,20) | The estimation was conducted through observing and simulating the daily movement paths of the staff within the operation area. The movement paths include high - frequency behaviors such as entering and exiting the operation room (door handle), accessing items (refrigerator handle), and moving equipment (stretcher handle). The behavioral research data of similar high - frequency contact surfaces (e.g., faucets) were referenced, and the conservative principle was adopted for setting. A triangular distribution was used to characterize the uncertainty of the estimation. |
| *F_counter_* | daily frequency of bare - handed contact with countertops | 8 times per day (Triangular distribution: 4, 6, 10) | Estimation is carried out based on structured task analysis and work simulation. By decomposing the typical operation process and combining on - site observation or expert interviews, the key nodes and frequencies of bare - hand contact with the tabletop are determined. The triangular distribution is used to characterize the uncertainty introduced by differences in work rhythm, task volume, and individual behavior variation. |
| *η_s-h_* | transfer efficiency from surfaces to hands | 0.20（Triangular distribution：0.10, 0.20, 0.35） | Refer to the study by Rusin et al. (2002) on the transfer efficiency of Gram-positive bacteria from non-porous surfaces to wet hands [28]. |
| *η_h-m_* | transfer efficiency from hands to mouth | 0.34（Triangular distribution：0.25, 0.34, 0.50） | In this model, the two-step microbial transfer efficiencies from the contaminated surface to the hand and then from the hand to the mouth were set by comprehensively referring to the experimental measurement data of Julian et al. (2010) [29] and the application parameters of the QMRA model of Ryan et al. (2014) [30], respectively. |
