# Supplementary material for: Assessing the risk of Staphylococcus aureus contamination and occupational exposure on high-frequency contact surfaces in funeral venues
Source: Front Public Health. 2026 Jun 5;14:1823786. doi: 10.3389/fpubh.2026.1823786 (PMC13279306; doi:10.3389/fpubh.2026.1823786)
Supplement: Supplementary file 2 [file Table_2.docx]

**Table 2 Overview of Comprehensive Data on Microbial Contamination**

|  | n | Total Bacterial Count （CFU/25cm^2^） | | | | | Number of qualified copies | Pass rate（%） | *P^a^* | *P^b^* | *S. aureus* | | Coliform bacteria |
| --- | --- | --- | --- | --- | --- | --- | --- | --- | --- | --- | --- | --- | --- |
|  |  | P5 | P25 | P50 | P75 | P95 |  |  |  |  | Detection Rate (%) | Concentration (MPN/cm²), P50(P25~P75) | Detection Rate (%) |
| Tools | 24 | 0 | 0 | 9.40 | 330.00 | 20,712.50 | 19 | 79.17 | 0.220 | 0.042 | 25.00 | 5.13(4.21 ~23.44) | 16.67 |
| Faucets | 13 | 0 | 0 | 2.60 | 75.40 | / | 11 | 84.62 |  |  | 13.04 | 23.25 (5~30) | 17.39 |
| Handles | 38 | 0 | 0 | 0 | 1.65 | 7.30 | 38 | 100 |  |  | 10.53 | 1.93 (1.06~2.64) | 17.95 |
| Counter | 10 | 0 | 0 | 0.10 | 0.70 | / | 10 | 100 |  |  | 10.00 | 1.85 | 30.00 |
| All Surfaces | 85 | 0 | 0 | 0 | 4.00 | 2,442.40 | 78 | 91.76 |  |  | 16.47 | 5.00(1.85~19.88) | 21.18 |

Note: P^a^ represents the chi-square test, and P^b^ represents the Kruskal-Wallis H test.
