# Supplementary material for: Assessing the risk of Staphylococcus aureus contamination and occupational exposure on high-frequency contact surfaces in funeral venues
Source: Front Public Health. 2026 Jun 5;14:1823786. doi: 10.3389/fpubh.2026.1823786 (PMC13279306; doi:10.3389/fpubh.2026.1823786)
Supplement: Supplementary file 3 [file Table_3.docx]

**Table 3 Monte Carlo Simulation Results (n=10,000)**

| **Statistical indicators** | **Scene A (Direct Exposure to Tools)** | **Scenario B (Indirect exposure to the environment)** | **Unit/Description** |
| --- | --- | --- | --- |
| *D_ingestion_*​ | | | |
| GSD | 65.8 (2.42) | 141.3 (2.51) | MPN/day |
| Median (95% CI) | 68.2 (31.5 – 156.3) | 146.7 (64.8 – 342.9) | MPN/day |
| Arithmetic mean (±SD) | 72.1 | 154.9 | MPN/day |
| Minimum ~ Maximum | 4.3~385.7 | 8.9 ~832.4 | MPN/day |
| RRI | | | |
| Median (95% CI) | 0.47 (0.22 ~ 0.93) | 1.00 (0.48 ~ 1.00) | Dimensionless |
| Probability of RRI < 0.5 | 53.2% | 0% |  |
| Risk -threshold analysis | | | |
| Probability of exceeding 100 MPN/day | 24.8% | 48.3% | Referenced to 100 MPN/day |

The values mentioned above are examples based on the median input value of the parameter. In the actual simulation, the distribution characteristics of the parameters were considered, and both the median and the 95% confidence interval (CI) were derived from the output distribution.
